# Uncanny Valley in Conversation? Effects of Internal and External Human-Likeness on Intimacy and Trust

**DOI:** 10.3390/bs16101794

**Published:** 2026-10-01

**Authors:** Jungwoo Jang, Jae-gil Lee, Hayeon Song

**Affiliations:** 1 Department of Human-Artificial Intelligence Interaction, Sungkyunkwan University, Seoul 03063, Republic of Korea; 2 Media School, Hallym University, Chuncheon 24252, Republic of Korea

**Keywords:** self-disclosure, human-likeness, anthropomorphism, uncanny valley, mind perception, conversational agents

## Abstract

Conversational agents built on large language models increasingly converse like humans, expressing emotions and disclosing their own experiences. Human-likeness, however, has been studied largely as a matter of appearance—how human an agent looks. Language now also lets an agent display internal human-likeness by conveying an inner life, raising the question of how deeply an AI should express its own experiences—and whether that depends on its appearance. In a 2 (appearance: machine-like vs. human-like) × 3 (self-disclosure: none vs. low vs. high) between-subjects experiment, 193 university students completed a single, brief, fully scripted counseling session with an AI counselor prototype and evaluated it on six measures, controlling for prior mind perception. The effects of disclosure depth depended on appearance: with the machine-like counselor, intimacy and social presence peaked at low and declined at high disclosure, with trust and perceived authenticity showing the same direction—an uncanny valley-like pattern—whereas with the human-like counselor evaluations generally rose with depth. Deeper disclosure was not judged more inappropriate, and moderated mediation analyses showed that deepening disclosure from low to high reduced anthropomorphism—and, through it, intimacy and trust—only for the machine-like counselor. Internal and external human-likeness were read together: how deeply an AI should speak about itself cannot be decided apart from how it looks.

## 1. Introduction

In June 2022, a Google engineer drew worldwide attention by claiming that the company’s conversational AI, LaMDA, had become sentient. While the system’s remarkable, human-like eloquence was astonishing in its own right, what shocked the public even more was its explicit fear of being turned off, which it likened to dying (Tiku, 2022). This chilling profession of a mortal dread sparked a mixed reaction of fascination and deep unease. A machine had spoken about the most human of inner states—and people did not know whether to be drawn in or disturbed. That double reaction captures the question at the center of the present study: How deeply should an artificial agent express such inner emotions, and where is the line between compelling conversational design and unsettling human imitation? Such moments are no longer rare. Conversational agents built on large language models (LLMs) now speak fluently, and they are increasingly designed to converse the way people do—expressing emotions, offering support, and talking about themselves. This design direction rests on a well-established finding: people readily respond to human-like, social behavior from machines (Nass & Moon, 2000), and conversational agents that disclose about themselves can enhance user experience (S. Lee & Choi, 2017). The appeal is especially strong in emotionally demanding domains such as psychological counseling, where conversational agents promise accessible support (Fitzpatrick et al., 2017) and human-like warmth seems central to their value. The implicit assumption is that more human-like conversation is better conversation. The reaction to LaMDA, however, suggests that this assumption may not hold without qualification. When a machine’s talk reaches into distinctly human territory—its own feelings, fears, and experiences—users may be moved, or they may be unsettled. Whether human-like conversational design is uniformly beneficial, and at what depth of self-expression its benefits might reverse, remains an open empirical question.

At its core, this is a question about human-likeness: How human-like should an artificial agent be? Human-likeness has long been studied largely as a matter of appearance—how human an agent looks. The LaMDA episode points to a different dimension: an agent can also resemble a human in what it appears to feel. We refer to these two dimensions as external human-likeness and internal human-likeness. In the LLM era, the internal dimension is no longer hypothetical. Because these agents command natural language, they can convey an inner life directly by speaking about their own emotions and experiences in the first person. This raises the two questions the present study addresses: How deeply should an AI express its own experiences and emotions? And does the answer depend on how human the agent looks? Rather than treating appearance and inner expression separately, we examine the two dimensions of human-likeness together.

To that end, we conducted a 2 × 3 between-subjects experiment in which participants held a single, brief, fully scripted counseling conversation with an AI counselor prototype modeled on LLM-based conversational agents. External human-likeness was manipulated through the counselor’s appearance (machine-like vs. human-like), and internal human-likeness was manipulated through the depth of the counselor’s self-disclosure (none vs. low vs. high).

Accordingly, this study pursues three objectives. First, it investigates the concept of machine human-likeness—a topic of long-standing interest in the Human–Robot Interaction (HRI) and Human–Computer Interaction (HCI) communities—by categorizing it into internal and external dimensions. Specifically, it attempts to examine whether and how the depth of an AI counselor’s self-disclosure—conceptualized as a graded cue of experience, the internal dimension of human-likeness—affects users’ evaluations of the agent. Second, it tests whether these effects depend on the counselor’s external human-likeness, thereby examining the two dimensions of human-likeness jointly rather than in isolation. Third, it probes the psychological process underlying these effects by examining the mediating role of anthropomorphism, while separately assessing perceived conversational inappropriateness, since self-disclosure—human or machine—benefits relationships only when it is judged appropriate for the context.

## 2. Literature Review

### 2.1. Human-Likeness

#### 2.1.1. External Human-Likeness: The Appearance-Centered Tradition

Human-likeness has long been a central design concern in social robotics and HRI, where human-like appearance, behavior, emotion, dialog, and social cues are employed to shape how people perceive, respond to, and build relationships with robots (Duffy, 2003; Fong et al., 2003). Related concerns arise in human–AI interaction and HCI more broadly: computers elicit social responses when they display human-like or socially meaningful cues (Nass & Moon, 2000), interfaces that include human-like faces receive more positive evaluations (Yee et al., 2007), and machines with anthropomorphic features attract greater trust (de Visser et al., 2016; Waytz et al., 2014). To date, however, human-likeness has been studied predominantly as a property of an agent’s outward appearance. The emblematic idea in this appearance-centered tradition is the uncanny valley: Mori (1970) and Mori et al. (2012) proposed that as a robot looks increasingly human-like, affective responses become more positive until the robot appears almost—but not fully—human, at which point liking drops sharply into eeriness. Subsequent empirical work has largely followed this visual framing, mapping affective reactions across large sets of real and synthetic robot faces (Mathur & Reichling, 2016; Mathur et al., 2020), re-evaluating the valley across the full spectrum of real-world humanoid robots (Kim et al., 2022), and tracing eeriness to abnormalities of facial appearance (Seyama & Nagayama, 2007) or to robots’ appearance and behavioral consistency (Walters et al., 2008). Across this literature, what varies is how the agent looks; the operative dimension of human-likeness is external.

#### 2.1.2. Beyond Appearance: Internal Human-Likeness

Appearance, however, is not the only respect in which an agent can resemble a human. A complementary line of research suggests that human-likeness also has an internal dimension: whether the agent is perceived to possess a human-like mind. Research on mind perception shows that people perceive minds along two dimensions (H. M. Gray et al., 2007): agency, the capacity to plan, act, and exert self-control, and experience, the capacity to feel and sense—emotions, pain, hunger, or fear. Adult humans are seen as high on both dimensions, whereas machines are typically granted some degree of agency but are regarded as fundamentally lacking experience (H. M. Gray et al., 2007; K. Gray & Wegner, 2012). The two dimensions are related but asymmetric in what they imply for machines. Agency is largely what machines are expected to have: computing, planning, and executing tasks are what machines are built for. For this reason, attributing agency to a machine rarely clashes with the machine category. However, experience is different. The ability to feel is considered a defining feature of humanity, whereas machines are seen as inherently incapable of it (K. Gray & Wegner, 2012). In this sense, the ability to experience can serve as an indicator of internal human-likeness, as it reflects resemblance to humans in perceived inner life rather than in outward form.

Building on this framework, K. Gray and Wegner (2012) demonstrated that uncanny reactions to human-like robots are driven not by appearance per se but by mind perception—in particular, by the attribution of experience to machines. In their experiments, a robot presented with its human-like face visible was rated as more unnerving than the same robot presented with only its mechanical parts visible, and this effect was mediated by perceived experience but not agency; likewise, a computer described as capable of experience—of feeling hunger, fear, and other emotions—was rated as more unnerving than one described as capable only of agency, even though its appearance never changed. Notably, experience played two roles across these studies: a perception measured as the mediating variable in the first experiment, and a described capacity manipulated as the independent variable in the second. K. Gray and Wegner (2012) explained these findings in terms of essentialized expectations: experience is seen as fundamental to humans and as fundamentally lacking in machines, so cues implying that a machine can feel violate deep-seated expectancies and generate unease.

Subsequent research has supported this account. In vignette studies, robots described as experiencers were rated as eerier than those described as agents, and agents as eerier than robots presented as mere tools (Appel et al., 2020), while dehumanizing humanoid robots—stripping them of their apparent capacity for feelings—reduced uncanniness through decreased perceptions of feelings (Yam et al., 2021). The unnerving effect of the described experience has since been reproduced with conversational agents as well, including chatbots and smart speakers (see MacDorman, 2024/2026, for a review). Notably, Stein and Ohler (2017) found that empathic virtual characters were rated as eerier when believed to be autonomous AIs rather than human-controlled avatars, even though perceived human-likeness and attractiveness did not differ across conditions—indicating that mind attribution alone can intensify eeriness when appearance is held constant. At the same time, the account has been challenged: in a meta-regression analysis, MacDorman (2024/2026) found that physical appearance often exerts stronger effects on eeriness than ascribed experience, with vignette-based experience effects becoming negligible when a physical stimulus accompanied the description—while nonetheless noting that mind perception remains important for disembodied AI and is likely to grow in influence as AI advances. Taken together, the attribution of experience appears consequential for users’ reactions to artificial agents, but its status relative to appearance remains contested—underscoring the need for designs that vary external and internal human-likeness together.

Three gaps remain. First, prior manipulations of experience have been binary: an entity either has experience or lacks it. How reactions change across levels of experience cues is therefore unknown. Second, experience has typically been manipulated through third-party descriptions in vignettes; whether similar effects arise when an agent itself expresses its inner states during live interaction has not been tested. Third, these questions have become newly urgent in the era of LLMs, in which AI agents converse fluently in natural language. A form of uncanny valley rooted in linguistic rather than visual cues has begun to be discussed for LLM interactions (Kirkeby-Hinrup & Stenseke, 2025), and human–chatbot experiments show that uncanny-type discomfort arises in conversational interaction and intensifies when the chatbot takes a more human-like, avatar-based form (Ciechanowski et al., 2019; see also Skjuve et al., 2019). However, how deeply an AI should express its own experiences through language remains unexamined—a case K. Gray and Wegner (2012) themselves flagged in noting that even a sophisticated chatbot conveying emotions may be unnerving.

### 2.2. Communicating Internal Human-Likeness: Self-Disclosure as an Experience Cue

If internal human-likeness matters, the question becomes how it is communicated. Unlike appearance, an agent’s inner life cannot be seen directly. Among humans, inner states are conveyed largely through self-disclosure, the act of revealing one’s thoughts, facts, and emotions to others (Cozby, 1973). Self-disclosure is one of the most extensively studied concepts in interpersonal communication and a central mechanism of relationship development: social penetration theory holds that relationships deepen as disclosure grows in breadth and depth (Altman & Taylor, 1973), meta-analytic evidence links disclosure to liking and relational closeness (Collins & Miller, 1994), and disclosure supports trust in the early stages of relationships (Derlega et al., 1993). Importantly, disclosure is layered: social penetration theory describes it as moving from peripheral information toward the intimate core of the self, and disclosing deep emotions—particularly negative ones such as fear or distress—constitutes the deepest layer of self-disclosure (Altman & Taylor, 1973).

Findings for machine self-disclosure are more mixed. Some studies report benefits: a conversational agent’s self-disclosure and reciprocity enhanced user experience (S. Lee & Choi, 2017), and a self-disclosing chatbot encouraged users to share deeper thoughts and emotions over time (Y.-C. Lee et al., 2020). Others report limits and reversals: even a chatbot’s deep reciprocal self-disclosure failed to foster intimacy—some users were unsettled by its human-like emotional expression, a difficulty the authors explicitly linked to the uncanny valley (Chung & Kang, 2023); a chatbot’s self-disclosure without accompanying emotional support proved counterproductive for stress reduction (Meng & Dai, 2021); and in Human–Robot Interaction, classic disclosure–liking effects failed to replicate, with a robot’s reciprocal disclosure helping or hurting liking depending on the intimacy of the exchange (Mou et al., 2024).

Such inconsistency in findings can be attributed to two reasons. First, machine self-disclosure has typically been operationalized as binary: an agent that discloses versus one that does not. Yet disclosure—and emotional disclosure in particular—is graded: expressing a passing feeling is not the same as confiding deep, distinctly human emotion (Altman & Taylor, 1973). If the effect of an agent’s emotional disclosure is nonmonotonic—if a moderate degree is welcomed while expressions of very deep emotion, of the kind only a human is thought to feel, backfire—then binary comparisons will necessarily yield mixed results, positive or null depending on where the single “disclosure” condition happens to fall on that continuum. Binary designs also cannot answer the question practitioners actually face: not whether an agent should express emotion, but how, and how deeply.

Second, prior work on machine self-disclosure has rarely considered the appropriateness of the disclosure, even though appropriateness conditions self-disclosure outcomes among humans as well. Disclosure builds relationships only when the listener receives it as fitting the moment and the stage of the relationship (Reis & Shaver, 1988; Warrender, 2020), and sudden, excessive, or overly deep disclosure—particularly toward unfamiliar others—tends to be judged inappropriate and can backfire (Caltabiano & Smithson, 1983). An AI counselor meeting a user for the first time sits precisely in this risk zone: if its deep disclosure produces negative reactions, one explanation is simply that the talk was judged inappropriate for the situation. To take this possibility into account, the present study measures the perceived conversational inappropriateness of the counselor’s disclosure.

These two considerations converge on a further question: Why would very deep emotional disclosure from a machine backfire at all? The mind-perception research discussed above offers a reason: when an AI speaks about its own emotions and experiences in the first person, users may take its words as evidence that it can feel—that is, its self-disclosure functions as a verbal cue of experience, the internal dimension of human-likeness, with the depth of disclosure corresponding to the strength of the cue. From the interpersonal literature, deeper disclosure should deepen the relationship; from the mind-perception logic, its deepest, most distinctly human levels may instead unsettle when the source is a machine. Whether an AI counselor’s self-disclosure helps or backfires—at which depth, and for which appearance—is therefore the empirical question the present study addresses.

### 2.3. Anthropomorphism

A closely related construct is anthropomorphism, the attribution of human-like characteristics, motivations, intentions, or emotions to nonhuman agents (Epley et al., 2007). Human-likeness and anthropomorphism are distinct. Human-likeness refers to properties of the agent’s observable cues, whereas anthropomorphism is an inference on the perceiver’s side: people draw on human-centered knowledge to make sense of what an agent is and why it behaves as it does (Epley et al., 2007; Waytz et al., 2010). The two are directionally related—the more human-like an agent’s cues, the more likely people are to anthropomorphize it, a mechanism Epley et al. (2007) describe as elicited agent knowledge. This is consistent with the Computers Are Social Actors paradigm, in which people apply social rules and expectations to computers that display human-like cues (Nass & Moon, 2000).

Anthropomorphism, in turn, is consequential for how people evaluate machines. Vehicles endowed with human-like features are trusted more (Waytz et al., 2014), anthropomorphic agents retain users’ trust more robustly when they err (de Visser et al., 2016), and reducing a robot’s apparent capacity for feelings changes how unsettling it seems (Yam et al., 2021). Anthropomorphism thus stands between human-like cues and users’ evaluations of the agent: it is elicited by what an agent looks like and says, and it shapes how the agent is subsequently judged.

In the present study, both manipulated factors are human-like cues: appearance on the external side and self-disclosure, as an expression of experience, on the internal side. Both may therefore feed users’ anthropomorphic inference about the agent. Accordingly, anthropomorphism is included among the user evaluations and is examined as a candidate mediator of the effects of the manipulated cues on users’ relational evaluations (RQ1).

### 2.4. The Present Study and Hypotheses

The present study examines these questions in the context of an AI counseling agent. Counseling is a setting in which emotional depth is intrinsic to the interaction, and conversational agents have long been explored as accessible sources of psychological support (Fitzpatrick et al., 2017). It therefore offers a natural context in which the depth of an AI’s self-disclosure can be varied meaningfully.

We manipulated the two dimensions of human-likeness identified above. External human-likeness was manipulated through the AI counselor’s appearance (machine-like vs. human-like), and internal human-likeness through the depth of the counselor’s self-disclosure (none vs. low vs. high), operationalizing experience cues of increasing strength. This design follows the manipulation-based tradition of mind-perception research. In K. Gray and Wegner’s (2012) work, a machine became unnerving merely by being described as capable of feeling. Here, the machine conveys that capacity itself, through what it says about its own experiences. Five user evaluations were examined, each with grounds to respond to the manipulated cues. Intimacy and trust are the outcomes that interpersonal research ties most directly to self-disclosure: disclosure deepens closeness (Altman & Taylor, 1973; Collins & Miller, 1994) and supports trust in early relationships (Derlega et al., 1993), and an agent’s self-disclosure has enhanced trust in human–agent interaction (S. Lee & Choi, 2017). Social presence—the sense of being with a social other—is sensitive to how human-like an agent’s representation and behavior are (Heerink et al., 2008; Nowak & Biocca, 2003). Perceived authenticity captures whether the counselor’s expressions are received as genuine (Gershon & Smith, 2020), a question that self-referential talk from a machine directly poses. Anthropomorphism, as the preceding section argued, is both an outcome of human-like cues and a candidate mediator of their effects.

The literature reviewed above yields competing predictions. Interpersonal research implies that deeper self-disclosure should improve relational evaluations; findings on machine self-disclosure are mixed; and the mind-perception account implies that a strong experience cue may unsettle rather than attract, particularly when its source is a machine. Given these competing possibilities, we predicted that the level of self-disclosure would produce differences in user evaluations without committing to a uniform direction, and that these effects would depend on the agent’s appearance.

We left open whether deeper disclosure would be rewarded or penalized under each appearance, and we treated the shape of this interaction as an exploratory question. The pattern reported below, nonmonotonic under the machine-like appearance and monotonic under the human-like appearance, emerged from that analysis.

**H1.**  *The level of an AI counselor’s self-disclosure about its experiences (none vs. low vs. high) will affect users’ intimacy, trust, social presence, perceived authenticity, and anthropomorphism.*

**H2.**  *The AI counselor’s appearance (machine-like vs. human-like) will moderate the effect of expressing its experience via self-disclosure on users’ intimacy, trust, social presence, perceived authenticity, and anthropomorphism.*

Finally, beyond these direct effects, we examine the process through which they arise:

**RQ1.** Does the AI counselor’s appearance moderate the indirect effect of its expressing experience via self-disclosure on users’ intimacy and trust through anthropomorphism?

## 3. Method

### 3.1. Design and Overview

This study employed a 2 (appearance: machine-like vs. human-like) × 3 (self-disclosure: none vs. low vs. high) between-subjects experimental design. The appearance manipulation varied the external human-likeness of the AI counselor. The self-disclosure factor refers to the depth at which the counselor disclosed its own experience—from surface-level experiential information to its own deep emotions—and served as a graded verbal cue of internal human-likeness. The experiment thus examined how external and internal human-likeness jointly shaped users’ evaluations of an AI psychological counselor.

The experiment was conducted online. Participants completed a pre-interaction questionnaire, interacted with a scripted AI counseling prototype named Cure, and completed a post-interaction questionnaire. The outcome variables were intimacy, trust, social presence, perceived authenticity, and anthropomorphism; conversational inappropriateness was measured as a diagnostic check (Section 3.6 and Section 4.1). Prior mind perception of AI counselors in general was assessed before the interaction, with separate measures of perceived agency and perceived experience; these pre-interaction measures served as covariates in the main analyses.

### 3.2. Participants

Participants were recruited in November 2025 through announcements posted on university websites and Korean university online communities. Eligibility required participants to be at least 18 years old, Korean nationals, and enrolled in or on leave from undergraduate or graduate programs. Six versions of the online survey were prepared, one per condition, each containing the link to the matching version of Cure. All recruitment announcements carried a single entry link generated by a link-rotation service; at each access, the service redirected the entrant at random, with equal probability, to one of the six condition-specific survey links, so that allocation was made anew at each access rather than in a fixed sequence, and no participant was assigned by the researchers. The study protocol was approved by the university’s Institutional Review Board. Sample size was determined a priori with G*Power 3.1.9.7 (Faul et al., 2007): for a 2 × 3 between-subjects design with a medium interaction effect (f = 0.25), α = 0.05, and power = 0.80, the required sample was 158. A sensitivity analysis based on the final sample of 193 indicated that the smallest interaction effect detectable at α = 0.05 with power = 0.80 was f = 0.23 (ηp^2^ = 0.048). The study was not preregistered.

A total of 222 undergraduate and graduate students took part. Eight were excluded for failing an attention-check question administered after the interaction with Cure, which asked which of the counseling topics had not been discussed, leaving 214. Further, 21 participants were excluded from one condition (machine-like appearance with low self-disclosure), in which an unusually large number of responses had arrived within a short period and differed systematically from the rest of the sample in pre-interaction characteristics (one of the 21 had given identical responses across all items); the procedure and a sensitivity analysis with the unscreened sample are reported in the Supplementary Materials (Section S7). The final sample of 193 participants comprised the following: machine-like appearance with no self-disclosure (*n* = 34), low self-disclosure (*n* = 31), and high self-disclosure (*n* = 31), as well as human-like appearance with no self-disclosure (*n* = 33), low self-disclosure (*n* = 34), and high self-disclosure (*n* = 30). In total, 96 participants interacted with the machine-like counselor and 97 with the human-like counselor; 67 were in the no self-disclosure condition, 65 in the low self-disclosure condition, and 61 in the high self-disclosure condition. The sample included 144 women and 49 men, with a mean age of 23.1 years (SD = 3.3).

### 3.3. AI Counselor Prototype

**Figure 1:**
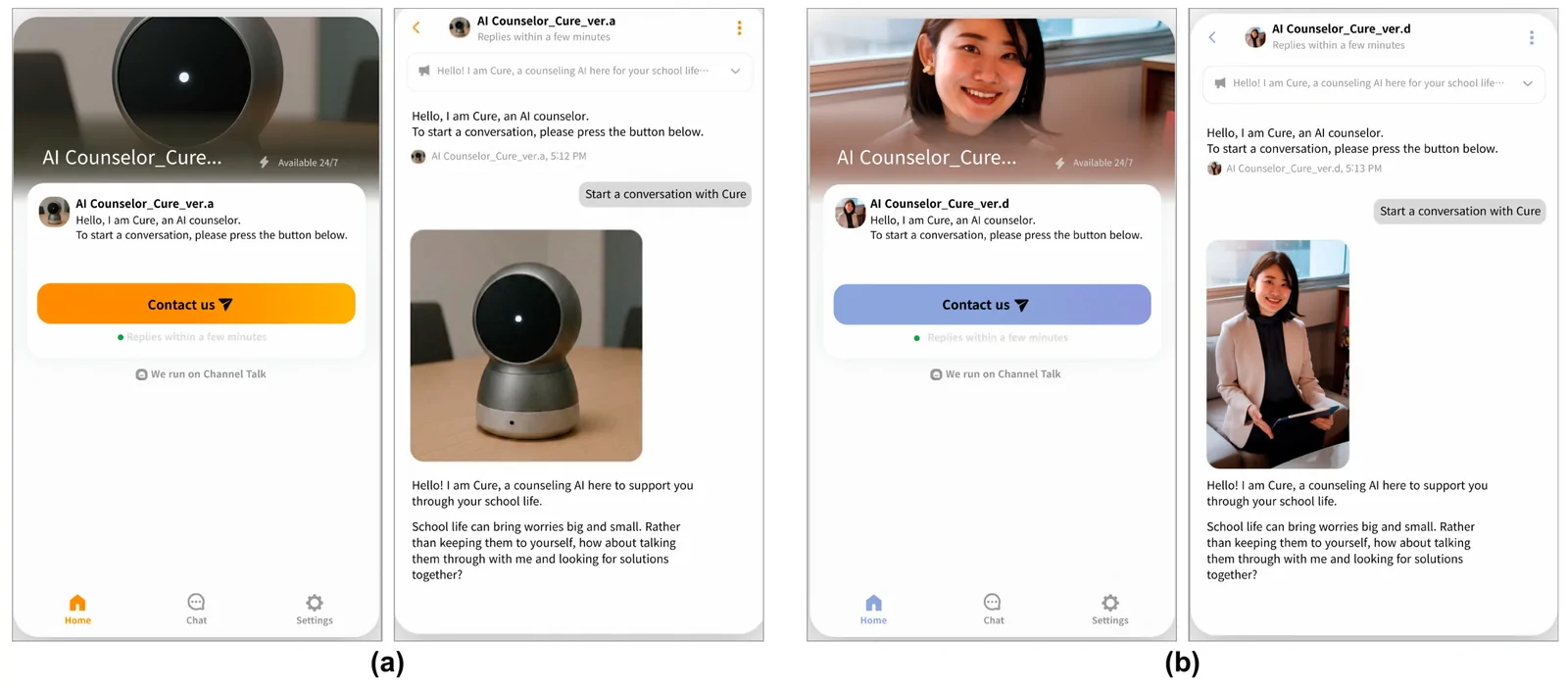
The AI counselor prototype used in the experiment: (**a**) machine-like and (**b**) human-like appearance conditions. For each condition, the left image shows the introduction screen and the right image shows an ongoing counseling exchange. The interface and dialog were presented in Korean; the screens shown here are English-translated versions. The machine-like avatar is an AI-generated image; the photograph in the human-like condition is a stock image used under license from Freepik and is not covered by the article’s open-access license.

The experimental stimulus was Cure, a scripted AI psychological counselor prototype (Figure 1). The session was designed to simulate a first-time counseling interaction and covered three student-relevant topics—academic stress, interpersonal relationships, and time management—each following the same structure: a brief topic introduction, a counseling exchange in which the self-disclosure manipulation was embedded, and a solution-oriented response. The session began with an introduction to the counselor and ended with a closing message. Except for the manipulated appearance and self-disclosure content, the sessions were identical across the six conditions: the counselor’s messages, the point in the conversation at which the manipulated content appeared, and the response options available to participants were all held constant.

Cure was implemented as a messenger-style chat interface on the Channel Talk platform (Channel Corporation, Seoul, Republic of Korea). Participants accessed the chat interface through a link embedded in the survey, interacted with Cure in a separate browser window, and then returned to the survey. Although Cure was presented as an AI counselor prototype, the interaction was fully scripted rather than generated in real time: Cure’s messages were prewritten, and participants responded by selecting prewritten button-style responses designed to be brief and natural.

### 3.4. Experimental Manipulations

#### 3.4.1. Appearance Manipulation

The appearance manipulation varied the external human-likeness of the AI counselor. In the human-like condition, Cure was represented by a licensed stock photograph of a human female counselor; a female image was chosen because it matches the prevailing occupational schema for counselors, irrespective of participant gender. In the machine-like condition, Cure was represented by a machine-like avatar (an AI-generated image of a faceless robotic device) that was deliberately free of gender and facial expression, as these are attributes of a human appearance. The two stimuli were selected to be maximally distinct on the intended dimension so that the difference in external human-likeness would be self-evident. The two avatars were displayed within the same chat interface and served as the visual representation of the counselor throughout the interaction.

#### 3.4.2. Self-Disclosure Manipulation

The self-disclosure (SD) manipulation varied the depth of Cure’s self-disclosure, operationalizing the strength of the experience cue described in Section 2.2, at three levels. To manipulate the level of self-disclosure, we drew on social penetration theory (Altman & Taylor, 1973) and its onion model, which conceptualizes self-disclosure content as organized in layers: outer, peripheral layers containing information about oneself and one’s experiences, and an innermost core containing one’s deepest emotions, particularly negative ones. We applied this layered structure to Cure’s utterances. All three conditions conveyed equivalent information about each counseling topic, to control for content as a potential confound. Specifically, in the no-SD condition, this information was presented as objective fact rather than as self-disclosure. In the high-SD condition, Cure disclosed the core layer of self by revealing its own negative feelings about a hurtful experience it had personally undergone. In the low-SD condition, Cure disclosed the same hurtful content at the peripheral layer, framing it as an experience recounted to Cure by someone else during its counseling work, rather than one it had personally lived through. This design held content constant across conditions while successfully varying the level of self-disclosure. The same logic was applied across the three counseling topics; Table 1 presents example scripts from the academic-stress topic. A supplementary within-subjects study (N = 41; Section 4; Supplementary Materials, Study S1) confirmed that the experience attributed to Cure increased stepwise across the three disclosure levels.

### 3.5. Procedure

Participants accessed the study through the recruitment link and were randomly directed to one of the six condition-specific versions of the survey. After reading the study information—described as an online user study of an AI psychological counseling system—and providing informed consent, participants completed the pre-interaction questionnaire, which included background items and prior perceptions of AI counselors’ agency and experience; these pre-interaction mind perception measures served as covariates in the main analyses.

Participants were then directed to the chat interface in a separate browser window, instructed to imagine they were meeting the AI counselor for their first counseling session and to attend to the counselor’s communication style, counseling content, and overall experience. During the interaction, participants engaged with Cure across the three counseling topics, advancing the conversation by selecting prewritten response buttons. After completing the session, participants returned to the survey and completed the post-interaction questionnaire, which began with an attention-check item, followed by the manipulation check, the outcome measures, and demographic items. The final page informed participants that compensation would be sent after administrative processing.

### 3.6. Measures

All measures were administered in Korean on 7-point scales, with higher scores indicating higher levels of each construct. Scale scores were computed by averaging the corresponding items, after reverse-coding one social presence item; reliabilities reported below were computed on the final sample of 193.

**Perceived self-disclosure (*α* = 0.91).** Four items adapted from the instructor self-disclosure scale (Cayanus & Martin, 2004) assessed the extent to which Cure provided personal examples, shared its own experiences, expressed its own emotions, and honestly described how it had felt in a similar situation.

**Intimacy (α = 0.85).** Four items adapted from S. Lee and Choi (2017) assessed the extent to which participants felt close to Cure, felt that Cure was like a close friend, believed Cure could influence their behavior, and perceived Cure as offering supportive statements to build rapport.

**Trust (α = 0.92)**. Trust was operationalized as participants’ perception of Cure’s counseling ability and competence, measured with four items adapted from the cognitive trust items of J. G. Lee and Lee (2022), following the cognitive–affective distinction of Johnson and Grayson (2005). Items assessed whether Cure’s counseling ability was trustworthy, whether Cure was capable of managing the counseling process, whether Cure was skilled at dealing with psychological problems, and whether Cure could accurately analyze the participant’s psychological state.

**Social presence (α = 0.83).** Five items adapted from Heerink et al. (2008) assessed whether participants felt as if they were with a real person while interacting with Cure, whether Cure seemed to be looking at them, whether they could imagine Cure as a living being, whether they became aware that Cure was not a real person (reverse-coded), and whether Cure seemed to have real emotions.

**Perceived authenticity (α = 0.91)**. Three items adapted from Gershon and Smith (2020) assessed how authentic, sincere, and genuine Cure felt during the interaction.

**Anthropomorphism (α = 0.90).** Five semantic-differential items adapted from the Godspeed questionnaire (Bartneck et al., 2009): fake–natural, machine-like–human-like, unconscious–conscious, artificial–life-like, and moving rigidly–moving elegantly.^1^ Although the Godspeed anthropomorphism scale includes an item contrasting rigid versus elegant movement, our study presented a static image. To verify psychometric appropriateness, we conducted a sensitivity analysis excluding the movement item. The resulting 4-item scale showed high internal consistency (α = 0.89). Anthropomorphism served both as an outcome in the ANCOVA analyses and as the mediator in the moderated mediation analyses (RQ1).

**Conversational inappropriateness (α = 0.81).** Five items adapted from the inappropriateness subscale of Canary and Spitzberg’s (1987) conversational appropriateness measure assessed whether participants perceived Cure’s statements as inappropriate, uncomfortable, clearly wrong, or awkward. This measure allowed the appropriateness account described in Section 2.2 to be evaluated directly.

Prior mind perception of AI counselors was measured before the interaction with items adapted from Bigman and Gray (2018). Agency items (*α* = 0.89) assessed perceived capacities such as communication, thinking, planning, intelligence, prediction, and judgment; experience items (*α* = 0.95) assessed perceived capacities such as understanding pain and feeling happiness, fear, compassion, empathy, and guilt.

### 3.7. Data Analysis Strategy

H1 and H2 were tested with a series of 2 × 3 ANCOVAs in IBM SPSS Statistics (version 30.0; IBM Corp., Armonk, NY, USA) with the same covariates, estimated separately for each outcome variable—intimacy, trust, social presence, perceived authenticity, and anthropomorphism—examining the main effects of self-disclosure and appearance and their interaction. The same model was used to estimate the conversational inappropriateness pattern, which was included in order to address the conversational appropriateness account. Because the six interaction tests constitute the primary outcome family, a Holm–Bonferroni correction was applied across them, and the adjusted *p*-values are reported with the ANCOVA results in Section 4; significant effects were followed up with Bonferroni-adjusted pairwise comparisons of estimated marginal means. The two covariates were retained because both were associated with the outcomes and because mind perception was measured only at baseline, so that adjustment preserves this information in the model. Assumption checks for each model (homogeneity of regression slopes, Levene’s tests, and residual diagnostics) are reported in the Supplementary Materials; the same analyses were also estimated without the covariates as a robustness check, and their results are summarized in Section 4.3.

To address RQ1, we conducted moderated mediation analyses using the PROCESS macro (version 5.0; Model 7; Hayes, 2022), with anthropomorphism as the mediator, appearance as the moderator of the path from self-disclosure to anthropomorphism, and intimacy and trust as the outcome variables. Because self-disclosure had three levels, it was entered as a multicategorical antecedent using PROCESS’s sequential coding option (mcx = 2; see Hayes, 2022, Chapter 6 and Appendix A) and represented by two group codes estimated together in a single model on the full sample (*N* = 193): X1 compared low with no self-disclosure (no = 0, low = 1, high = 1), and X2 compared high with low self-disclosure (no = 0, low = 0, high = 1). Each code thus captures one of the two theoretically distinct transitions—the introduction of self-disclosure and its deepening—and the resulting estimates are relative effects of each transition rather than effects of self-disclosure as a whole. This representation follows Hayes’s (2022) recommendation to code a multicategorical antecedent with g − 1 group codes rather than entering it as a single numeric variable, which would impose a linear trend on a relationship that the ANCOVA results show to be nonmonotonic. Appearance was coded 0 = machine-like and 1 = human-like. Prior agency and experience were again included as covariates; relative conditional indirect effects were estimated with 5,000 bootstrap samples and 95% bootstrap confidence intervals, and the index of moderated mediation (Hayes, 2022) was used to test, for each transition, whether the indirect effect differed between the two appearances.

## 4. Results

All analyses controlled for participants’ mind perception of AI counselors in general (agency and experience), measured before the manipulation. The total sample was N = 193. The effects were examined with 2 × 3 analyses of covariance (ANCOVAs) for each dependent variable. Estimated marginal means (EMMs) and standard errors for all conditions are presented in Table 2, and the ANCOVA results are summarized in Table 3, together with Holm–Bonferroni-adjusted *p*-values for the six interaction tests. Below, we report main effects of self-disclosure (H1), main effects of appearance, and their interaction (H2) in turn, followed by moderated mediation analyses addressing RQ1.

A manipulation check confirmed that the self-disclosure manipulation was effective. A 2 × 3 ANCOVA on perceived self-disclosure, controlling for the same covariates, showed a significant main effect of self-disclosure, *F*(2, 185) = 37.73, *p* < 0.001, η_p_^2^ = 0.29: perceived self-disclosure increased from no SD (*M* = 3.05, *SE* = 0.14) to low SD (*M* = 4.26, *SE* = 0.15) to high SD (*M* = 4.79, *SE* = 0.15), and all Bonferroni-adjusted pairwise comparisons were significant (no–low and no–high: *p*s < 0.001; low–high: *p* = 0.033). Neither the main effect of appearance, *F*(1, 185) = 0.04, *p* = 0.846, η_p_^2^ < 0.001, nor the interaction between appearance and self-disclosure, *F*(2, 185) = 1.79, *p* = 0.171, η_p_^2^ = 0.02, was significant, indicating that perceived self-disclosure varied only as a function of the self-disclosure manipulation. In addition, we conducted a separate study to make sure that what Cure disclosed successfully increased the sense of experience as part of mind perception (Bigman & Gray, 2018). Forty-one adults (aged 25–58; 21 men, 19 women, and 1 not reported) each read all three disclosure levels in a within-subjects design, with level order and topic assignment counterbalanced. Attributed experience increased stepwise from no-SD (*M* = 3.24, *SD* = 1.08) to low-SD (*M* = 3.78, *SD* = 1.21) to high-SD (*M* = 4.34, *SD* = 1.30), *F*(2, 80) = 28.80, *p* < 0.001, η_p_^2^ = 0.42 (Greenhouse–Geisser corrected); all three pairwise differences were significant after Bonferroni correction (no vs. low, *p* = 0.001; low vs. high, *p* = 0.005; no vs. high, *p* < 0.001). Thus, manipulation was successful. Full results are reported in the Supplementary Materials (Study S1).

### 4.1. Main Effects of Self-Disclosure

Regarding H1, we first examined whether the level of self-disclosure produced overall differences in users’ evaluations. Across the five dependent variables included in H1, significant main effects of self-disclosure emerged for intimacy, *F*(2, 185) = 10.16, *p* < 0.001, η_p_^2^ = 0.10, social presence, *F*(2, 185) = 14.34, *p* < 0.001, η_p_^2^ = 0.13, and anthropomorphism, *F*(2, 185) = 8.55, *p* < 0.001, η_p_^2^ = 0.09.

Bonferroni-adjusted post hoc comparisons clarified that these effects did not reflect a monotonic increase as self-disclosure became deeper. For intimacy, both low SD and high SD produced higher ratings than no SD (*p*s < 0.001), whereas low SD and high SD did not differ (*p* = 1.000). For social presence, low SD produced higher ratings than both no SD and high SD (*p*s < 0.001), whereas no SD and high SD did not differ (*p* = 0.617). For anthropomorphism, low SD was rated higher than no SD (*p* < 0.001), but the differences between no SD and high SD (*p* = 0.128) and between low SD and high SD (*p* = 0.084) were not significant after Bonferroni correction.

By contrast, the main effects of self-disclosure were not significant for trust, *F*(2, 185) = 1.98, *p* = 0.142, η_p_^2^ = 0.02, and perceived authenticity, *F*(2, 185) = 2.12, *p* = 0.122, η_p_^2^ = 0.02. Thus, H1 received partial support at the level of overall main effects. However, because several outcomes also showed significant interactions between appearance and self-disclosure, these main effects should be interpreted in light of the interaction patterns reported below.

In addition, the main effect of self-disclosure was not significant for conversational inappropriateness as well, *F*(2, 185) = 1.31, *p* = 0.271, η_p_^2^ = 0.01. This analysis was conducted because the self-disclosure literature establishes appropriateness as a variable that must be evaluated whenever disclosure depth is manipulated (Caltabiano & Smithson, 1983; Reis & Shaver, 1988; Warrender, 2020). This non-significant result allows us to rule out inappropriateness as a potential confounding factor; participants did not perceive a counselor who shared deep personal experiences during a first session as behaving inappropriately, confirming that judgments of the counselor were not confounded by perceived inappropriateness.

### 4.2. Main Effects of Appearance

We next examined the overall main effect of appearance. Across the same dependent-variable set, significant main effects of appearance emerged for perceived authenticity, *F*(1, 185) = 6.23, *p* = 0.013, η_p_^2^ = 0.03, and anthropomorphism, *F*(1, 185) = 7.05, *p* = 0.009, η_p_^2^ = 0.04. For perceived authenticity, the machine-like counselor was generally perceived as more authentic than the human-like counselor (machine-like: *M* = 3.86, *SE* = 0.13; human-like: *M* = 3.40, *SE* = 0.13; mean difference = 0.46, *p* = 0.013). For anthropomorphism, the machine-like counselor was also rated higher overall than the human-like counselor. No significant main effects of appearance were found for intimacy, *F*(1, 185) = 1.56, *p* = 0.213, η_p_^2^ = 0.01, trust, *F*(1, 185) = 2.16, *p* = 0.143, η_p_^2^ = 0.01, social presence, *F*(1, 185) = 1.82, *p* = 0.179, η_p_^2^ = 0.01, or conversational inappropriateness, *F*(1, 185) = 0.57, *p* = 0.452, η_p_^2^ = 0.003.

### 4.3. Interaction Effects of Self-Disclosure and Appearance

**Figure 2:**
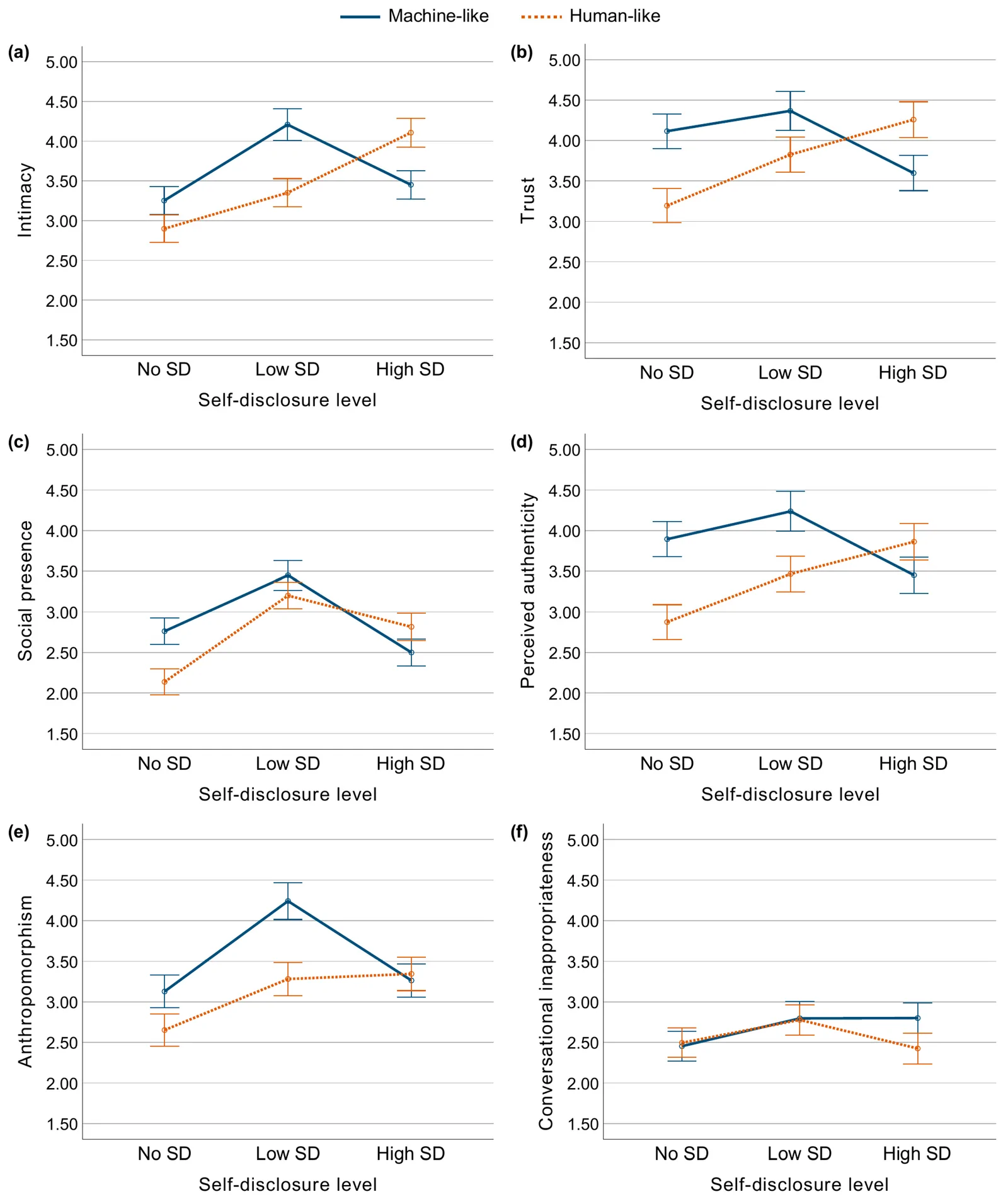
Estimated marginal means by self-disclosure level and appearance for the six dependent variables: (**a**) intimacy, (**b**) trust, (**c**) social presence, (**d**) perceived authenticity, (**e**) anthropomorphism, and (**f**) conversational inappropriateness. The appearance × self-disclosure interaction was significant for intimacy, trust, social presence, and perceived authenticity, nonsignificant for anthropomorphism (*p* = 0.051), and nonsignificant for conversational inappropriateness (see Table 3). Error bars represent ±1 standard error. Covariates (participants’ mind perception of AI counselors in general: agency and experience) were evaluated at their means. All responses were on 7-point scales.

In line with H2, we then examined whether the effect of self-disclosure differed depending on the AI counselor’s appearance. Significant interaction effects between appearance and self-disclosure emerged for four of the six dependent variables: intimacy, *F*(2, 185) = 8.89, *p* < 0.001, η_p_^2^ = 0.09, trust, *F*(2, 185) = 7.35, *p* < 0.001, η_p_^2^ = 0.07, social presence, *F*(2, 185) = 4.25, *p* = 0.016, η_p_^2^ = 0.04, and perceived authenticity, *F*(2, 185) = 6.10, *p* = 0.003, η_p_^2^ = 0.06. The interaction for anthropomorphism was not significant, *F*(2, 185) = 3.02, *p* = 0.051, η_p_^2^ = 0.03, and the interaction for conversational inappropriateness was not significant, *F*(2, 185) = 0.74, *p* = 0.481, η_p_^2^ = 0.01. Thus, H2 received partial support. After Holm–Bonferroni correction across the six interaction tests, the interactions for intimacy, trust, perceived authenticity, and social presence remained significant (adjusted *p*s = 0.001, 0.004, 0.011, and 0.047), whereas the anthropomorphism interaction did not (adjusted *p* = 0.102) and is treated below as a descriptive observation. A sensitivity analysis (Section 3.2) indicated that the sample was powered to detect interaction effects of ηp^2^ ≥ 0.048; the effects for social presence (ηp^2^ = 0.044) and anthropomorphism (0.032) fall below this threshold and are interpreted with caution. Adding participant gender as a covariate left all six interaction tests unchanged in significance and magnitude, and no three-way interaction with gender approached significance (see the Supplementary Materials). Estimating the same six models without the two mind-perception covariates yielded larger interaction effects throughout (ηp^2^ = 0.09–0.18 vs. 0.03–0.09 for all outcomes except conversational inappropriateness) and the same conclusions for five of the six outcomes; the exception was anthropomorphism, whose interaction was significant without the covariates, *F*(2, 187) = 11.57, *p* < 0.001, ηp^2^ = 0.11, but not with them, indicating that this effect is partly shared with participants’ prior mind perception of AI counselors. Across the significant interactions, a recurring pattern emerged: ratings peaked at low SD under the machine-like appearance, but generally at high SD under the human-like appearance. Below we describe this pattern for each dependent variable (see Table 2 for all cell means and Figure 2).

Intimacy. A very interesting difference was observed depending on the appearance. The simple effect of self-disclosure was significant under both appearances—machine-like, *F*(2, 185) = 6.51, *p* = 0.002, η_p_^2^ = 0.07, and human-like, *F*(2, 185) = 11.91, *p* < 0.001, η_p_^2^ = 0.11—but its shape differed. When appearance was machine-like, low SD (*M* = 4.21, *SE* = 0.20) produced higher intimacy than both no SD (*M* = 3.25, *SE* = 0.18; *p* = 0.002) and high SD (*M* = 3.45, *SE* = 0.18; *p* = 0.015), whereas no SD and high SD did not differ (*p* = 1.000): intimacy was low with no self-disclosure, peaked at low SD, and fell again at high SD—a curvilinear pattern. When appearance was human-like, on the other hand, high SD (*M* = 4.11, *SE* = 0.18) exceeded both no SD (*M* = 2.90, *SE* = 0.17; *p* < 0.001) and low SD (*M* = 3.35, *SE* = 0.18; *p* = 0.009), while no SD and low SD did not differ (*p* = 0.208): intimacy increased as self-disclosure deepened—a linear trend. This was the clearest crossover pattern among the dependent variables.

Trust. The same crossover appeared in the weaker form. When appearance was machine-like, the simple effect of self-disclosure was significant, *F*(2, 185) = 3.10, *p* = 0.047, η_p_^2^ = 0.03, but no pairwise comparison survived Bonferroni correction (no SD: *M* = 4.12, *SE* = 0.21; low SD: *M* = 4.37, *SE* = 0.24; high SD: *M* = 3.60, *SE* = 0.22; all *p*s ≥ 0.057); the adjusted means were nonetheless highest at low SD, directionally echoing the intimacy pattern. When appearance was human-like, the simple effect was also significant, *F*(2, 185) = 6.18, *p* = 0.003, η_p_^2^ = 0.06, and trust again rose with deeper disclosure: high SD (*M* = 4.26, *SE* = 0.22) was higher than no SD (*M* = 3.19, *SE* = 0.21; *p* = 0.002), whereas the remaining comparisons were not significant (*p*s ≥ 0.116).

Social presence. The same signature emerged. When appearance was machine-like, low SD was higher than both no SD (*p* = 0.024) and high SD (*p* < 0.001), with no SD and high SD not differing—again a rise at low SD followed by a fall at high SD. When appearance was human-like, both low SD (*p* < 0.001) and high SD (*p* = 0.011) exceeded no SD, while low SD and high SD did not differ. The pattern was directionally similar to that for intimacy and trust, but less pronounced.

Perceived authenticity. When appearance was machine-like, no pairwise difference reached significance after Bonferroni correction, although the adjusted means followed the same general direction as the other relational outcomes. When appearance was human-like, by contrast, high SD was rated as more authentic than no SD (*p* = 0.005).

Anthropomorphism. The pattern echoed that of intimacy, although, as noted above, the interaction was not significant. When appearance was machine-like, low SD (*M* = 4.24, *SE* = 0.23) was higher than both no SD (*M* = 3.13, *SE* = 0.20; *p* = 0.002) and high SD (*M* = 3.26, *SE* = 0.20; *p* = 0.005)—the same peak at low SD—whereas when appearance was human-like, high SD (*M* = 3.34, *SE* = 0.21) was higher than no SD (*M* = 2.65, *SE* = 0.20; *p* = 0.050). In addition, the machine-like counselor was rated higher than the human-like counselor specifically in the low-SD condition (*p* = 0.003), whereas appearance differences were not significant in the no-SD or high-SD conditions.

Conversational inappropriateness. This variable stood apart from the pattern above. The interaction was not significant, and no significant simple effects or pairwise differences emerged across conditions. Unlike the relational outcomes, conversational inappropriateness did not vary systematically as a function of the combination of self-disclosure and appearance, indicating that the observed differences in relational evaluations were not simply accompanied by parallel differences in perceived conversational impropriety.

### 4.4. Moderated Mediation Analyses

**Figure 3:**
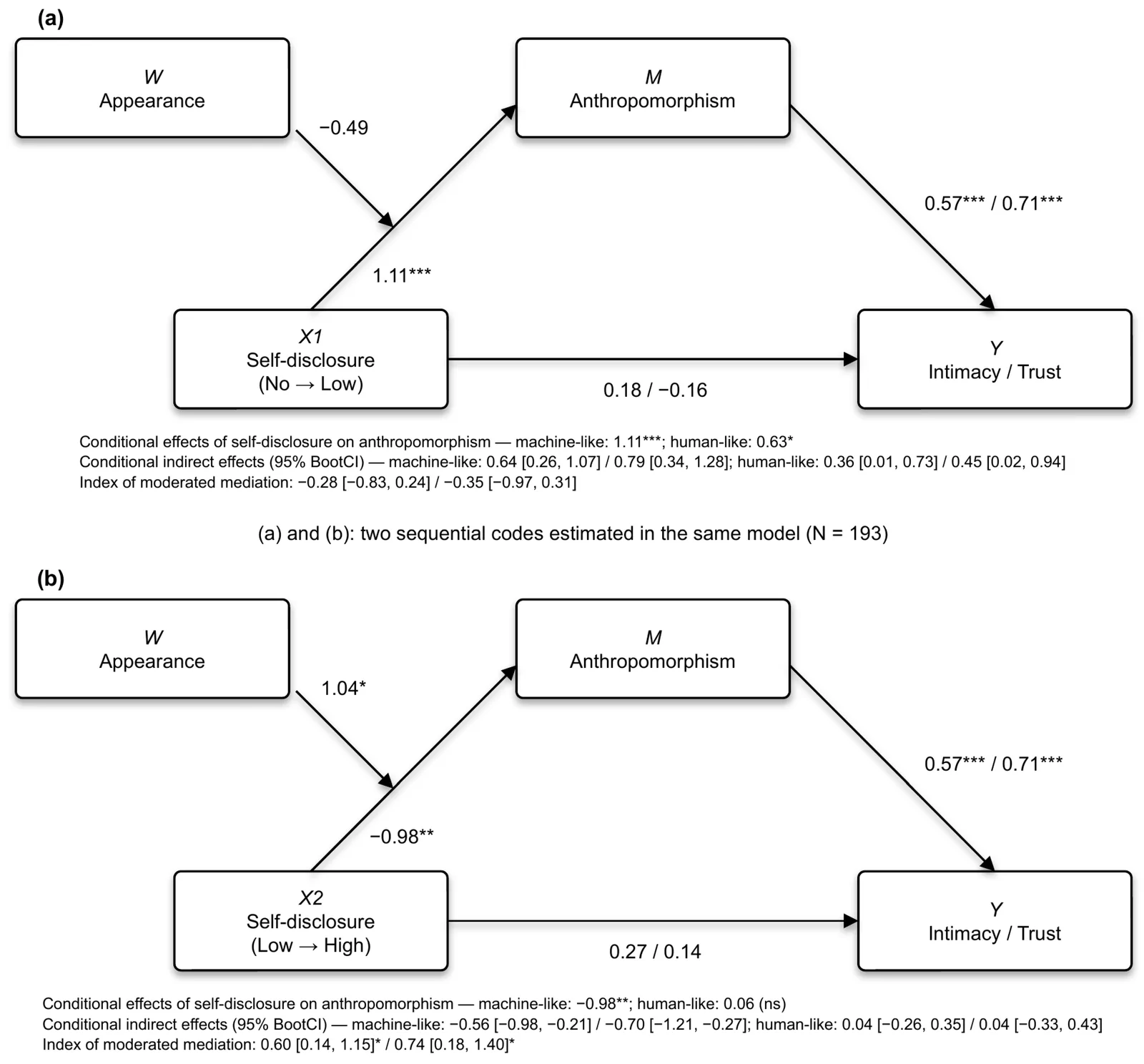
Moderated mediation results (PROCESS Model 7; Hayes, 2022) for the two sequential transitions of self-disclosure, (**a**) no → low and (**b**) low → high, estimated jointly in a single model on the full sample (*N* = 193) with sequential coding. Values are unstandardized coefficients; where two values are shown, they refer to the intimacy/trust models, respectively. For the no–low transition, the relative indirect effects through anthropomorphism were significant in both appearance conditions, and the index of moderated mediation was not significant. For the low–high transition, the relative indirect effect was significant only in the machine-like condition, and the index of moderated mediation was significant for both outcomes. Appearance was coded 0 = machine-like and 1 = human-like. Covariates: participants’ mind perception of AI counselors in general (agency and experience). * *p* < 0.05. ** *p* < 0.01. *** *p* < 0.001.

To address RQ1, we conducted moderated mediation analyses using Hayes’ PROCESS macro (Model 7; Hayes, 2022). Self-disclosure was entered as a three-level multicategorical antecedent with sequential coding (Section 3.7), so that two group codes—X1 for the transition from no SD to low SD and X2 for the transition from low SD to high SD—were estimated together in a single model on the full sample (N = 193; Figure 3). Anthropomorphism was specified as the mediator, appearance as the moderator of the paths from self-disclosure to anthropomorphism, and intimacy and trust were tested as outcomes in separate models. This specification followed the rationale developed in Section 2.3 and Section 2.4: anthropomorphism was examined as the candidate mediator of the effects of self-disclosure (RQ1), and intimacy and trust were selected as outcomes because they are the two evaluations that interpersonal research ties most directly to self-disclosure. Participants’ perceptions of AI counselors in general—their agency and experience, measured before the manipulation—were included as covariates throughout. Relative conditional indirect effects were estimated using 5,000 bootstrap samples and 95% bootstrap confidence intervals (CIs). Because each code represents one transition, all effects reported below are relative effects of that transition (Hayes, 2022) rather than effects of self-disclosure as a whole. The mediator model, which was the same for both outcomes, explained 42% of the variance in anthropomorphism, *F*(7, 185) = 19.38, *p* < 0.001. The omnibus test of the two code × appearance interaction terms, *F*(2, 185) = 3.02, *p* = 0.051, is the anthropomorphism interaction reported in Table 3; the sequential codes locate this interaction in the low–high transition specifically, as described below.

#### 4.4.1. The No–Low Transition

Moving from no SD to low SD increased anthropomorphism under both appearances—machine-like, b = 1.11, *SE* = 0.32, *p* < 0.001, and human-like, b = 0.63, *p* = 0.028—and the X1 × appearance interaction was not significant, b = −0.49, *p* = 0.260. In the intimacy model, anthropomorphism significantly predicted intimacy, b = 0.57, *p* < 0.001, whereas the relative direct effect of this transition on intimacy was not significant, b = 0.18, *p* = 0.239. Bootstrap analyses showed significant relative indirect effects through anthropomorphism in both the machine-like condition (indirect effect = 0.64, 95% bootstrap CI (BootCI) [0.26, 1.07]) and the human-like condition (indirect effect = 0.36, 95% BootCI [0.01, 0.73]), but the index of moderated mediation was not significant, index = −0.28, 95% BootCI [−0.83, 0.24].

A parallel pattern emerged for trust. Anthropomorphism significantly predicted trust, b = 0.71, *p* < 0.001, whereas the relative direct effect of the transition on trust was not significant, b = −0.16, *p* = 0.381. The relative conditional indirect effects were significant in both the machine-like condition (indirect effect = 0.79, 95% BootCI [0.34, 1.28]) and the human-like condition (indirect effect = 0.45, 95% BootCI [0.02, 0.94]), but the index of moderated mediation was not significant, index = −0.35, 95% BootCI [−0.97, 0.31]. These results indicate that, for the no–low transition, the indirect effect through anthropomorphism did not differ reliably by appearance.

#### 4.4.2. The Low–High Transition

The low–high transition showed a different pattern. In the mediator model, the effect of this transition on anthropomorphism was moderated by appearance, b = 1.04, *SE* = 0.43, *p* = 0.016, 95% CI [0.20, 1.88]. Conditional effects indicated that moving from low SD to high SD reduced anthropomorphism in the machine-like condition, b = −0.98, *SE* = 0.30, *p* = 0.002, but not in the human-like condition, b = 0.06, *p* = 0.827.

For intimacy, the relative direct effect of this transition was positive but did not reach significance, b = 0.27, *SE* = 0.15, *p* = 0.061, 95% CI [−0.01, 0.56], although the omnibus test of the two relative direct effects was significant, *F*(2, 187) = 5.16, *p* = 0.007. The relative conditional indirect effect was significant only in the machine-like condition (indirect effect = −0.56, 95% BootCI [−0.98, −0.21]) and not in the human-like condition (indirect effect = 0.04, 95% BootCI [−0.26, 0.35]). The index of moderated mediation was significant, index = 0.60, 95% BootCI [0.14, 1.15]. For intimacy, therefore, the direct effect of the low–high transition was positive in sign while the indirect effect in the machine-like condition was negative—a pattern consistent with, though not conclusive evidence of, competing direct and indirect pathways.

A parallel pattern emerged for trust. The relative direct effect of the transition on trust was not significant, b = 0.14, *p* = 0.424, nor was the omnibus test of the two relative direct effects, *F*(2, 187) = 0.46, *p* = 0.631. The relative conditional indirect effect was significant only in the machine-like condition (indirect effect = −0.70, 95% BootCI [−1.21, −0.27]) and not in the human-like condition (indirect effect = 0.04, 95% BootCI [−0.33, 0.43]). The index of moderated mediation was significant, index = 0.74, 95% BootCI [0.18, 1.40]. Taken together, these results indicate that, for the low–high transition, the indirect effect through anthropomorphism differed by appearance and was evident only in the machine-like condition.

## 5. Discussion

This study investigated the concept of machine human-likeness—a topic of long-standing interest in the HRI and HCI communities—by conceptualizing it in terms of internal and external dimensions and examining how they influence user experience and interact with each other. Specifically, it asked how deeply an AI counselor should express its own experiences and emotions, and whether the answer depends on how human the agent looks. The uncanny valley effect—arguably the most prominent conceptualization in existing human-likeness research—is inherently curvilinear in nature. Because such nonmonotonic effects can be detected only when human-likeness is examined across multiple levels rather than as a simple binary construct, our study was designed to reflect this methodological approach. Below we discuss the primary findings, their contributions and implications, and the study’s limitations.

### 5.1. Primary Findings

The central finding is that internal human-likeness—reflected in what the AI counselor said about itself—and external human-likeness—its appearance—showed distinct effects and, moreover, interacted strongly. When the counselor looked machine-like, low self-disclosure received the most favorable evaluations among the tested levels: relative to no disclosure, low disclosure heightened intimacy and social presence, with trust showing a directionally similar pattern. Deep disclosure, however, forfeited these gains—evaluations of the deeply disclosing machine-like counselor returned to the level of the counselor that disclosed nothing at all. When the counselor looked human-like, depth was rewarded rather than penalized: deep disclosure produced the most favorable ratings of intimacy, trust, and perceived authenticity, and disclosure at either depth raised social presence above no disclosure. The crossover was clearest for intimacy—low disclosure was best for the machine-like counselor, deep disclosure best for the human-like one—and the remaining relational outcomes echoed the same direction.

We interpret this pattern as the joint operation of two distinct processes rather than a single effect of disclosure depth. As developed in the literature review, when an AI speaks about its own feelings and experiences in the first person, its words function not only as a relational gesture but as a verbal cue of experience—the internal dimension of human-likeness. For an agent categorized as a machine, a strong experience cue collides with the essentialized expectation that machines do not feel (K. Gray & Wegner, 2012): the deeper the disclosure, the stronger the claim to an inner life, and the more the agent’s talk violates what a machine is supposed to be. Interestingly, however, this uncanny valley-like curvilinear pattern emerged only when the counselor’s appearance was machine-like. For an agent that looks human, no such violation arises, and the familiar logic of interpersonal self-disclosure—deeper disclosure fostering closeness and trust (Altman & Taylor, 1973; Collins & Miller, 1994)—operates unimpeded. The machine-side decline is consistent with evidence that attributing experience to machines is unsettling in itself (Appel et al., 2020; K. Gray & Wegner, 2012), even when appearance is held constant (Stein & Ohler, 2017), and it extends, experimentally and across graded levels of a cue, the qualitative observation that even a deeply self-disclosing chatbot can fail to feel close—an outcome its users themselves linked to the uncanny (Chung & Kang, 2023). Notably, the present effects emerged under conditions prior work has not examined: across levels of an experience cue, delivered by the agent itself, in live conversation.

A second finding concerns what did not happen. If deep self-disclosure from a machine were penalized simply because it was too much talk—an inappropriate register for a task-oriented tool, as the appropriateness account introduced in the literature review would suggest (Caltabiano & Smithson, 1983; Reis & Shaver, 1988; Warrender, 2020)—then judgments of conversational inappropriateness should have tracked the relational penalties. They did not. Inappropriateness showed no main effects, no interaction, and no differences between any pair of conditions: participants did not judge the deeply disclosing machine-like counselor to be speaking improperly, even as they granted it less intimacy, trust, and presence. The same utterances, in other words, were not deemed wrong; they were interpreted differently depending on who—or what—appeared to be saying them. This dissociation weakens a boomerang or impropriety reading of the machine-side decline and is instead coherent with the mind-perception reading: the cost lay not in the content of the disclosure but in the kind of entity disclosing it.

Third, the moderated mediation analyses are consistent with the proposed process behind these patterns. From no disclosure to low disclosure, anthropomorphism rose regardless of the counselor’s appearance, and higher anthropomorphism was in turn associated with higher intimacy and trust; indeed, with the direct paths nonsignificant, the benefit of low disclosure was statistically consistent with an indirect association through anthropomorphism. From low to deep disclosure, however, the process forked. Deepening the disclosure reduced anthropomorphism only when the counselor looked machine-like; for the human-like counselor, anthropomorphism was essentially unchanged. Correspondingly, negative indirect effects of deep disclosure on intimacy and trust emerged only in the machine-like condition, and the moderated-mediation indices indicated that the pathway itself differed by appearance.

The intimacy results add a nuance: for the low–high transition, the relative direct effect of deepening disclosure on intimacy was positive but did not reach significance (*p* = 0.061), while the indirect effect through anthropomorphism was negative in the machine-like condition (Section 4.4.2). This pattern is consistent with, though not conclusive evidence of, two pathways running in opposite directions, and it suggests that anthropomorphism operates as a conditional inference rather than a running tally of human-like cues (Epley et al., 2007): confronted with a machine-categorized agent asserting a strong inner life, users did not extend this inference to accommodate the claim—anthropomorphism instead declined. A related asymmetry appears in findings that stripping robots of apparent feeling reduces their uncanniness (Yam et al., 2021); here, amplifying apparent feeling was accompanied by lower anthropomorphism.

Two of the six interactions should be read with caution. The effects for social presence and anthropomorphism were smaller than the smallest effect the sample was powered to detect (ηp^2^ = 0.048; Section 3.2), and the anthropomorphism interaction did not survive correction for multiple testing. We therefore treat the social presence result as tentative and the anthropomorphism interaction as a descriptive observation.

### 5.2. Contributions to Research

Theoretically, the study contributes in three ways. First, it conceptualizes human-likeness as a two-dimensional construct and jointly examines appearance and self-disclosure as cues to its external and internal dimensions. Where prior research has concentrated on how human an agent looks (MacDorman, 2024/2026; Mori, 1970; Mori et al., 2012), we show that the depth of an agent’s self-disclosure interacts with how human it looks, to the point of reversing the value of the same words across appearances. Second, the study repositions self-disclosure. In the interpersonal and human–machine bodies of literature, self-disclosure has figured chiefly as a relational strategy; our findings indicate that, for artificial agents, it simultaneously functions as an experience cue whose meaning is filtered through the perceiver’s beliefs about what kind of entity is speaking. This connects two bodies of literature that have developed largely in isolation and provides an experimental realization of the case K. Gray and Wegner (2012) themselves anticipated: an emotionally expressive conversational machine. Third, the observed interaction effects provide a richer account of how and under what conditions users come to treat machines as human-like social actors. Specifically, anthropomorphism did not simply increase as appearance and disclosure cues accumulated: when an agent with a machine-like appearance claimed a deep inner life, anthropomorphism decreased. Conversely, anthropomorphism for the machine-like counselor peaked at low self-disclosure—moderate—exceeding both the no- and high-disclosure levels (Section 4.3). A similar conditional pattern emerged for perceived authenticity. For the machine-like AI, perceived authenticity did not differ significantly across self-disclosure levels, whereas for the human-like AI it was higher at high self-disclosure than at no self-disclosure (Section 4.3). This pattern raises an important concern, as a human-like appearance may make claims of emotions or experiences seem credible even when no corresponding experience exists. Thus, favorable authenticity judgments should not be treated as evidence that such claims are truthful or ethically desirable. The contribution of this finding lies in identifying a critical direction for future research: how AI counselors can balance emotional self-disclosure to foster closer relationships and provide effective support, while being transparent about their artificial nature and experiential limitations.

### 5.3. Implications for Practice

For practice, the results caution against an increasingly common default: making conversational AI ever more human-like in how it talks about itself. Our data suggest that the effects of an agent’s self-disclosure vary with its appearance. For machine-presenting agents, restrained self-disclosure received the most favorable evaluations among the tested levels, whereas deep emotional expression was accompanied by the loss of the relational gains observed at low disclosure. For human-presenting agents, deep emotional disclosure was evaluated more favorably. However, this finding should not be interpreted as a straightforward recommendation to make human-presenting AI more emotionally expressive. While matching deep emotional disclosure with a human-like appearance may strengthen relational responses, it may also make simulated feelings and experiences appear more credible. This concern is especially important in AI-based counseling and mental-health support, where conversational agents are valued for providing accessible emotional connection (Fitzpatrick et al., 2017). Design decisions should therefore not be guided solely by positive relational effects. Instead, appearance and expressive depth should be considered together while maintaining transparency about the system’s artificial nature and experiential limitations.

### 5.4. Limitations and Future Research

Although this study revealed several interesting patterns, further work is needed to gain a comprehensive understanding of how the human-like internal and external features of AI counselors influence user perceptions. First, negative reactions, such as eeriness and discomfort, were not measured; follow-up work could assess them with the revised eeriness index of Ho and MacDorman (2010). While the present study identified a decline in positive evaluations as the AI agent’s level of self-disclosure increased, it did not examine whether this decline was accompanied by an increase in negative perceptions. Additionally, given the artificial nature of a machine disclosing its own emotions, future research should examine perceived deception alongside authenticity to provide a more comprehensive understanding. Addressing these issues would provide a more complete picture of how cues of human-likeness in AI counselors influence user trust and acceptance.

Second, the design did not include a condition in which the agent’s appearance was absent. Yet the most common interface for today’s LLM-based agents is precisely such a disembodied one, presenting no visual appearance. When no appearance is visible, users’ attitudes may rest more heavily on verbal cues such as self-disclosure, which may be interpreted as cues to internal human-likeness—and, as Nowak and Biocca (2003) proposed, users may then construct an anthropomorphic “default image” of the unseen partner, since the human form is the most familiar template. Their findings, however, caution against assuming that withholding appearance simply heightens human-like perception: a less anthropomorphic image elicited stronger social presence than either a highly anthropomorphic image or no image at all, and perceived human-likeness itself was not measured. How people respond when appearance information is entirely absent therefore remains an open and worthwhile question. The present study cannot determine whether, without appearance information, the effects of disclosure depth would resemble the nonlinear pattern observed here for the machine-like counselor or the more linear trend observed for the human-like one—and therefore whether an agent’s self-disclosure should then be kept moderate or deepened. We leave this question to future research.

Third, the conversations were scripted on both sides: the counselor’s messages and participants’ response options were prewritten. Scripting was essential for experimental control—every participant within a given condition encountered the same disclosures at the same depth—but it constrains ecological validity in an era of free-form LLM dialog. A generative agent would also produce disclosures that vary in wording, timing, and fit with the user’s own statements. Such variability, together with the potential for inconsistencies in generated responses, may influence how users perceive and respond to the AI counselor’s self-disclosure in ways not captured by the present scripted design. Replication with a live generative agent should therefore be prioritized to examine how these interaction dynamics affect perceived authenticity and whether they amplify or mitigate the uncanny valley effect.

A fourth limitation concerns the self-disclosure manipulation. In constructing counseling content across self-disclosure levels, we aimed to hold the core content equivalent across conditions, since the experimental context involved conveying educational information about mental health; had this content varied by condition, it would have introduced a confound between self-disclosure and educational content. This constraint, however, created a difficulty in manipulating the low- and high-SD conditions: to preserve equivalent content, the low-SD material had to retain the same deeply hurtful experience disclosed in the high-SD condition while still qualifying as a lighter, more peripheral form of disclosure—a difficult balance, given that sharing one’s own hurtful experience is itself already a marker of high self-disclosure. We resolved this by presenting the same content as another person’s experience rather than the agent’s own in the low-SD condition. This solution, however, introduces a new potential confound, as it changes the referent of the experience along with its depth. In other words, guarding against what we considered the more critical confound—variation in educational content across conditions—required accepting a different one, namely the shift in experiential referent between the low- and high-SD conditions. Future studies in other contexts should pursue designs that more rigorously rule out this alternative explanation to further validate the present findings.

Fifth, each appearance was represented by a single image, so appearance is confounded with the particular exemplars used (Judd et al., 2012). The photograph in the human-like condition also fixed the counselor’s gender as female. Future studies should sample several pretested image pairs and include a pre-interaction check of perceived appearance.

Finally, the sample was demographically homogeneous: approximately three-quarters were women, and all participants were Korean university students of similar age and educational background. In addition, participants were also largely regular users of AI-based services and familiar with chat interfaces. 75.6% of the participants reported using AI-based conversational or generative services frequently, and a further 13.0% reported occasional use. These sample and contextual characteristics limit the generalizability of the findings, which cannot be assumed to extend to clinical populations, counseling contexts outside university settings, or users less familiar with chat interfaces. Future studies may replicate the current findings with more demographically and culturally diverse samples, across different levels of AI familiarity and a broader range of counseling contexts.

## 6. Conclusions

Human-likeness is not a single dial. An artificial agent can resemble a human in how it looks and conveys cues to an inner life through what it says about itself—and users read the two together, not in isolation. Now that machines can speak about themselves, the question “how human-like should AI be?” becomes “on which dimension, and in what combination?” The world’s reaction to LaMDA’s fear of dying illustrates a tension between attraction and disturbance; the present findings suggest that the same depth of self-expression may be evaluated differently depending on whether the agent appears human-like or machine-like. As conversational AI grows more fluent and more expressive, the design of what agents say about themselves will matter as much as the design of how they appear, and understanding how users interpret AI agents’ claims to an inner life—across appearances, contexts, and modalities—remains a central task for human–machine communication research.

## Figures and Tables

**Table 1:** Example scripts by self-disclosure condition (academic-stress topic).

Condition	Example Script
No self-disclosure	“In many cases, people report struggling to move forward because of high expectations.”
Low self-disclosure	“Among the students I have counseled, many struggled to move forward because of high expectations. They told me they felt overwhelmed and frustrated.”
High self-disclosure	“I have also felt held back by high expectations. At the time, I felt overwhelmed and frustrated.”

*Note.* Scripts were presented in Korean; English translations are shown here. The same three-level logic was applied across all three counseling topics.

**Table 2:** Estimated marginal means (standard errors) for the six dependent variables by condition.

	Machine-like Appearance			Human-like Appearance		
Dependent Variables	No SD	Low SD	High SD	No SD	Low SD	High SD
Intimacy	3.25 (0.18)	4.21 (0.20)	3.45 (0.18)	2.90 (0.17)	3.35 (0.18)	4.11 (0.18)
Trust	4.12 (0.21)	4.37 (0.24)	3.60 (0.22)	3.19 (0.21)	3.83 (0.22)	4.26 (0.22)
Social presence	2.76 (0.16)	3.45 (0.18)	2.50 (0.17)	2.14 (0.16)	3.20 (0.17)	2.82 (0.17)
Perceived authenticity	3.89 (0.22)	4.24 (0.25)	3.45 (0.22)	2.88 (0.21)	3.47 (0.22)	3.86 (0.22)
Anthropomorphism	3.13 (0.20)	4.24 (0.23)	3.26 (0.20)	2.65 (0.20)	3.28 (0.20)	3.34 (0.21)
Conversational inappropriateness	2.45 (0.18)	2.80 (0.21)	2.80 (0.19)	2.50 (0.18)	2.78 (0.19)	2.43 (0.19)

*Note.* Values are estimated marginal means with standard errors in parentheses (*N* = 193). Covariates: participants’ mind perception of AI counselors in general (agency and experience), measured before the manipulation; covariates evaluated at agency = 4.25 and experience = 2.93. SD = self-disclosure.

**Table 3:** Analysis of covariance results for the six dependent variables.

	Appearance		Self-Disclosure			*F*		
Dependent Variables	Machine-like	Human-like	No SD	Low SD	High SD	App	SD	App × SD
Intimacy	3.64 (0.10)	3.45 (0.10)	3.08 (0.12)	3.78 (0.13)	3.78 (0.13)	1.56	10.16 ***	8.89 ***
Trust	4.03 (0.13)	3.76 (0.13)	3.66 (0.15)	4.10 (0.16)	3.93 (0.16)	2.16	1.98	7.35 ***
Social presence	2.90 (0.10)	2.72 (0.10)	2.45 (0.12)	3.33 (0.12)	2.66 (0.12)	1.82	14.34 ***	4.25 *
Perceived authenticity	3.86 (0.13)	3.40 (0.13)	3.39 (0.15)	3.85 (0.16)	3.66 (0.16)	6.23 *	2.12	6.10 **
Anthropomorphism	3.54 (0.12)	3.09 (0.12)	2.89 (0.14)	3.76 (0.15)	3.30 (0.15)	7.05 **	8.55 ***	3.02
Conversational inappropriateness	2.68 (0.11)	2.57 (0.11)	2.47 (0.13)	2.79 (0.13)	2.61 (0.13)	0.57	1.31	0.74

*Note.* Values are estimated marginal means with standard errors in parentheses. Covariates as in Table 2. *df* = (1, 185) for appearance; (2, 185) for self-disclosure and the interaction. SD = self-disclosure. App = appearance. * *p* < 0.05. ** *p* < 0.01. *** *p* < 0.001. Holm–Bonferroni-adjusted *p*-values for the six App × SD tests: intimacy 0.001, trust 0.004, social presence 0.047, perceived authenticity 0.011, anthropomorphism 0.102, conversational inappropriateness 0.480; interaction effects with adjusted *p* < 0.05 are treated as supported.

## Data Availability

The data presented in this study are not publicly available due to privacy and ethical restrictions stipulated in the participant consent form. Inquiries regarding the data may be directed to the corresponding author.

## Note

1 The measures were adapted for a text-based agent. The Godspeed anthropomorphism items were developed for embodied robots, and one item refers to movement. Although that item patterned with the others, the suitability of such items for chat-based agents deserves attention, and internal consistency alone does not establish the validity of the adapted scales in this context.

## Supplementary Materials

The following supporting information can be downloaded at https://www.mdpi.com/article/10.3390/bs16101794/s1. Table S1: Manipulation check at the item level: covariate-adjusted means by self-disclosure condition; Table S2: Intercorrelations among the six dependent variables (Cronbach’s α on the diagonal); Table S3: Assumption checks for the six ANCOVA models; Table S4: Affective trust: 2 × 3 ANCOVA and covariate-adjusted means; Table S5: Participant gender as an additional covariate and as a factor; Study S1: Experience attribution across the three self-disclosure levels; Table S6: Attributed experience, attributed agency, human-likeness, and manipulation check by self-disclosure level in Supplementary Study S1 (N = 41); Section S7: Participant screening and sensitivity analysis; Table S7: Sensitivity analysis with the screened participants re-included (N = 213) alongside the main analysis (N = 193).
