# Supplementary material for: Uncanny Valley in Conversation? Effects of Internal and External Human-Likeness on Intimacy and Trust
Source: Behav Sci (Basel). 2026 Oct 1;16(10):1794. doi: 10.3390/bs16101794 (PMC13701690; doi:10.3390/bs16101794)
Supplement: Supplementary file 1 [file behavsci-16-01794-s001.zip › behavsci-4504866-supplementary.pdf]

Supplementary Materials for: *Uncanny Valley in Conversation? Effects of Internal and External Human-Likeness on Intimacy and Trust*

Jungwoo Jang [1], Jae-gil Lee [2] and Hayeon Song [1,*]

[1] Department of Human-Artificial Intelligence Interaction, Sungkyunkwan University, Seoul, Republic of Korea

[2] Media School, Hallym University, Chuncheon, Gangwon, Republic of Korea

* Correspondence: songhy@skku.edu

All analyses use the final analytic sample (N = 193; machine-like n = 96, human-like n = 97; no SD n = 67, low SD n = 65, high SD n = 61). Unless noted, models include participants' prior mind perception of AI counselors (agency, experience) as covariates, as in the main text.

Table S1. Manipulation check at the item level: covariate-adjusted means by self-disclosure condition

Item (perceived self-disclosure)	No SD	Low SD	High SD	No vs. Low	Low vs. High	No vs. High
1. Cure gives personal examples in its responses	3.61	4.68	4.64	< .001	1.000	< .001
2. Cure shares its own experiences in its responses	2.87	4.42	4.96	< .001	.088	< .001
3. Cure reveals its own feelings in its responses	2.86	3.94	4.64	< .001	.024	< .001
4. Cure honestly expressed how it had felt in similar situations	2.85	3.98	4.92	< .001	< .001	< .001

Note. Estimated marginal means from 2 (appearance) × 3 (self-disclosure) ANCOVAs on each item, with covariates evaluated at agency = 4.25 and experience = 2.93; p values are Bonferroni-adjusted pairwise comparisons. The appearance × self-disclosure interaction was non-significant for all four items (ps ≥ .115).

Table S2. Intercorrelations among the six dependent variables (Cronbach's α on the diagonal)

	1	2	3	4	5	6
1. Intimacy	(.85)					
2. Cognitive trust	.81***	(.92)				
3. Social presence	.76***	.65***	(.83)			
4. Perceived authenticity	.79***	.84***	.72***	(.91)		
5. Anthropomorphism	.79***	.77***	.77***	.85***	(.90)	
6. Conversational inappropriateness	−.17*	−.25***	−.01	−.18*	−.13	(.81)

Note. Pearson correlations, N = 193. * $p < .05$. *** $p < .001$. Reliabilities were computed on the final sample.

Table S3. Assumption checks for the six ANCOVA models

Dependent variable	Levene's test F(5, 187), p	Shapiro–Wilk on standardized residuals W, p	Skewness / kurtosis of residuals	Homogeneity of regression slopes (covariate × factor terms)
Intimacy	2.04, .075	.989, .163	−.11 / .81	Self-disclosure × experience $F(2, 179) = 4.77$, $p = .010$; other terms $p \geq .212$
Cognitive trust	4.11, .001	.989, .152	.25 / .73	Self-disclosure × agency $F(2, 179) = 4.24$, $p = .016$; other terms $p \geq .164$
Social presence	3.06, .011	.993, .555	.19 / .10	All terms $p \geq .111$

Perceived authenticity	2.24, .053	.995, .771	.15 / −.03	All terms p ≥ .172
Anthropomorphism	1.82, .112	.994, .657	.06 / −.36	All terms p ≥ .303
Conversational inappropriateness	2.59, .027	.942, < .001	1.07 / 2.37	All terms p ≥ .293

Note. Residuals were taken from the reported model (two covariates, appearance, self-disclosure, and their interaction). Homogeneity of regression slopes was tested by adding the four covariate × factor terms to the reported model. Because cell sizes were nearly equal (30–34), the F tests are robust to the heterogeneity of variance observed for cognitive trust, social presence, and conversational inappropriateness. For conversational inappropriateness, whose residuals departed from normality, a Kruskal–Wallis test across the six cells confirmed the null result, $H(5) = 7.54$, $p = .183$. Where the homogeneity-of-slopes assumption was not met (intimacy, cognitive trust), the unadjusted analyses summarized in Section 4.3 of the main text yield the same conclusions.

Table S4. Affective trust: 2 × 3 ANCOVA and covariate-adjusted means

Effect	F	df	p	ηp^2
Appearance	0.02	1, 185	.883	.000
Self-disclosure	4.14	2, 185	.017	.043
Appearance × self-disclosure	0.81	2, 185	.445	.009
**Covariate-adjusted means**	**No SD**	**Low SD**	**High SD**	
Machine-like	3.82	4.28	4.18	
Human-like	3.63	4.20	4.54	
Marginal	3.73	4.24	4.36	

Note. Affective trust was measured with four items adapted from the affective trust items of Lee and Lee (2022), following Johnson and Grayson (2005), $\alpha = .92$. Marginal comparisons (Bonferroni): no vs. high p = .020, no vs. low p = .103, low vs. high p = 1.000. Within the human-like appearance, no vs. high p = .017; within the machine-like

appearance, no pairwise difference was significant (ps ≥ .624). Levene's test $F(5, 187) = 4.32$, $p < .001$; cell sizes were nearly equal.

Table S5. Participant gender as an additional covariate and as a factor

Dependent variable	Appearance × SD with gender as covariate		Gender (covariate)		Appearance × SD × gender	
	F-value	ηp^2	F-value	ηp^2	F-value	ηp^2
Intimacy	8.70***	.086	0.27	.001	0.35	.004
Cognitive trust	7.59***	.076	1.08	.006	0.14	.001
Social presence	4.67*	.048	2.07	.011	0.79	.009
Perceived authenticity	6.48**	.066	2.87	.015	0.13	.001
Anthropomorphism	2.73	.029	2.37	.013	0.02	.000
Conversational inappropriateness	1.00	.011	5.63*	.030	1.73	.019

Note. The F-value and ηp^2 columns under "Appearance × SD with gender as covariate" and "Gender (covariate)" are from 2 (appearance) × 3 (self-disclosure) ANCOVAs in which participant gender was added as a third covariate alongside prior mind perception (agency and experience), $F(2, 184)$ and $F(1, 184)$, respectively; the columns under "Appearance × SD × gender" are from 2 (appearance) × 3 (self-disclosure) × 2 (gender) ANOVAs estimated without covariates and report the three-way interaction, $F(2, 181)$. Gender coded 1 = male (n = 49), 2 = female (n = 144). The three-way models are exploratory given the small number of male participants per cell and are estimated without covariates; in these models the appearance × self-disclosure interaction remained significant for the five evaluative outcomes (ps ≤ .006) and non-significant for conversational inappropriateness ($p = .354$). * $p < .05$. ** $p < .01$. *** $p < .001$.

Supplementary Study S1. Experience attribution across the three self-disclosure levels

Purpose. The manipulation check in the main study established that participants perceived increasing self-disclosure across the no-, low-, and high-SD conditions, but it did not directly test whether the three levels differ in the inner experience they cue. In response to the reviewers' concern that the low-SD condition might function as a cue of professional expertise rather than as a weaker cue of experience, we conducted a small within-subjects stimulus-evaluation study after receiving the reviews.

Participants. Forty-one adults (aged 25–58 years, $M = 35.3$, $SD = 6.6$; 21 men, 19 women, 1 not reported) were recruited on 5–6 September 2026 by convenience sampling: the first author posted a call for participation in personal and laboratory group chats on a messaging app (KakaoTalk), a family member of the first author reposted the same call in their own group chats, and volunteers who contacted the first author received an individual survey link, responded anonymously, and were compensated with a KRW 5,000 gift voucher. No participant was excluded: all completed the three blocks, and no response preceded the start of distribution. Three respondents whose ratings did not vary within at least one block were retained in the main analysis and removed in a sensitivity analysis.

Materials and design. Each participant read three excerpts of Cure's counseling scripts from the main study, one for each counseling topic (academic stress, interpersonal relationships, time management), each presented at a different self-disclosure level (no-SD, low-SD, high-SD). The order of the three levels (six sequences) and the assignment of topics to levels (three assignments) were counterbalanced across 18 codes, so that every participant saw each level once and each topic once, and each level appeared equally often at each serial position and with each topic. After each excerpt, participants rated the experience they attributed to Cure (the six experience items of the mind-perception measure used in the main study; $\alpha = .89$), the agency they attributed to Cure (six items; $\alpha = .92$), Cure's human-likeness on a single machinelike–humanlike item (1–7), and a manipulation-check item ("Cure honestly expressed how it had felt in similar situations"; 1–7).

Analysis. For each measure we ran a one-way repeated-measures ANOVA on self-disclosure level with Greenhouse–Geisser correction and Bonferroni-corrected pairwise comparisons, and repeated the tests with Friedman and Wilcoxon signed-rank tests as robustness checks. Serial position and topic were examined as nuisance factors.

Results. Table S6 reports the means, standard deviations, and tests. Attributed experience increased stepwise across the three levels, $F(2, 80) = 28.80$, $p < .001$, $\eta p^2 = .42$ ($\varepsilon = .92$), and all three pairwise differences were significant (no < low, $p = .001$, $dz = 0.64$; low < high, $p = .005$, $dz = 0.53$; no < high, $p < .001$, $dz = 1.26$); the Friedman test agreed, $\chi^2(2) = 33.50$, $p < .001$. Attributed agency showed a smaller effect, $F(2, 80) = 4.64$, $p = .015$, $\eta p^2 = .10$, with only the no–high difference significant after correction ($p = .002$). Ratings of human-likeness also increased across levels, $F(2, 80) = 8.04$, $p < .001$, $\eta p^2 = .17$, with only the no–high difference significant ($p < .001$). The manipulation-check item replicated the stepwise pattern found in the main study, $F(2, 80) = 14.64$, $p < .001$, $\eta p^2 = .27$, with all pairwise differences significant ($ps \leq .045$). Neither serial position ($p = .32$) nor topic ($p = .97$) affected attributed experience. Removing the three flagged respondents ($N = 38$) left every result unchanged (experience: $\eta p^2 = .44$; all pairwise $ps \leq .005$).

Conclusion. The three disclosure levels differ in the experience participants attribute to Cure, and low-SD occupies an intermediate position between no-SD and high-SD rather than functioning only as a cue of professional expertise; attributed agency did not show the same graded pattern.

Limitations. Participants were a convenience sample drawn from the first author's personal and laboratory networks (including a call reposted by a family member), so some were acquainted with the research team and the sample is not representative of the university population of the main study; because the design was within-subjects, such between-person differences do not confound the comparisons among disclosure levels. Participants also read the scripts rather than interacting with Cure, and rated human-likeness with a single item.

Table S6. Attributed experience, attributed agency, human-likeness, and manipulation check by self-disclosure level in Supplementary Study S1 ($N = 41$).

Measure	No-SD M (SD)	Low-SD M (SD)	High-SD M (SD)	$F(2, 80)$	p	ηp^2	No vs. Low	Low vs. High	No vs. High

Attributed experience (6 items)	3.24 (1.08)	3.78 (1.21)	4.34 (1.30)	28.80	< .001	.42	.001	.005	< .001
Attributed agency (6 items)	4.77 (1.20)	4.98 (1.34)	5.22 (1.30)	4.64	.015	.10	.515	.470	.002
Human-likeness (1 item)	3.27 (1.52)	3.73 (1.69)	4.27 (1.82)	8.04	< .001	.17	.228	.124	< .001
Manipulation check (1 item)	3.61 (1.77)	4.37 (1.77)	5.24 (1.48)	14.64	< .001	.27	.045	.007	< .001

Note. Within-subjects design; all ratings on 7-point scales. F tests are Greenhouse–Geisser corrected (uncorrected df shown); pairwise columns give Bonferroni-corrected p values (three comparisons per measure). The manipulation-check item was "Cure honestly expressed how it had felt in similar situations."

S7. Participant screening and sensitivity analysis

Procedure. Of the 222 participants recruited, 8 failed the attention-check question, leaving 214. One participant gave the same response to every item and was excluded as a straight-liner. Participants had been assigned to conditions by a link rotator that distributed the six condition-specific survey links with equal probability; however, 33 of the 52 responses in the machine-like, low-SD condition arrived within a two-hour window on a single evening, and these respondents differed systematically from the rest of the sample (nearly all reported no prior use of AI services, had been recruited outside the host university, and reported markedly higher pre-interaction attitudes toward AI). Within this condition, the 20 respondents with the highest composite pre-interaction attitude scores (AI literacy, machine heuristic, and mind perception–agency and –experience, standardized within the condition), among the 27 who reported no prior AI use and had been recruited outside the host university, were excluded, bringing the cell to a size comparable to the other cells. All 21 exclusions were therefore in the machine-like, low-SD condition; the final sample of 193 comprised 34, 31, and 31 participants in the machine-like no-, low-, and high-SD conditions and 33, 34, and 30 in the corresponding human-like conditions. Because the screening was applied after data collection and drew on variables related to the covariates, every analysis was re-run with the 20 screened participants re-included (N = 213); Table S7 reports the results alongside the main analysis.

Table S7. Sensitivity analysis with the screened participants re-included (N = 213) alongside the main analysis (N = 193).

	Main analysis (N = 193; machine-like, low-SD n = 31)				Re-included (N = 213; n = 51)			
**Appearance × self-disclosure interaction**	**F(2, 185)**	**p**	**ηp^2**	**Holm p**	**F(2, 205)**	**p**	**ηp^2**	**Holm p**

Intimacy	8.890	< .001	.088	.001	10.551	< .001	.093	< .001
Cognitive trust	7.353	< .001	.074	.004	8.167	< .001	.074	.002
Social presence	4.250	.016	.044	.047	4.741	.010	.044	.029
Perceived authenticity	6.102	.003	.062	.011	6.823	.001	.062	.006
Anthropomorphism	3.024	.051	.032	.102	4.039	.019	.038	.038
Conversational inappropriateness	.736	.480	.008	.480	.990	.373	.010	.374
**Machine-like cell means, M (SE)**	**No SD**	**Low SD**	**High SD**		**No SD**	**Low SD**	**High SD**	
Intimacy	3.25 (.18)	4.21 (.20)	3.45 (.18)		3.38 (.17)	4.37 (.16)	3.58 (.17)	
Cognitive trust	4.12 (.21)	4.37 (.24)	3.60 (.22)		4.24 (.21)	4.48 (.20)	3.72 (.21)	
**Index of moderated mediation, low → high transition (X2 × appearance)**	**Index**		**95% bootstrap CI**		**Index**		**95% bootstrap CI**	
Intimacy	.596		[.142, 1.154]		.644		[.181, 1.213]	
Cognitive trust	.741		[.176, 1.405]		.790		[.231, 1.456]	

Note. Both columns use the model reported in the main text (covariates: prior mind perception–agency and –experience, evaluated at their sample means). Holm correction was applied across the six interaction tests (Holm p = Holm-adjusted p). Main-analysis values are those reported in Tables 2–3 and Section 4.4; the N = 213 analyses used PROCESS Model 7 with sequential coding of self-disclosure, 5,000 bootstrap samples, and a fixed random seed (20260906). The N = 213 sample excludes only the straight-lining respondent.
